# An Oral Small Molecule VEGFR2 Inhibitor, Apatinib, in Patients with Recurrent or Refractory Cervical Cancer: A Real World Study

**DOI:** 10.1155/2020/3852373

**Published:** 2020-06-22

**Authors:** Ning Li, Ziyi Wang, Guangwen Yuan, Yangchun Sun, Rong Zhang, Xiaoguang Li, Nan Li, Jing Wang, Lingying Wu

**Affiliations:** ^1^Department of Gynecologic Oncology, National Cancer Center/Cancer Hospital, Chinese Academy of Medical Sciences and Peking Union Medical College, Beijing 100021, China; ^2^Department of Gynecologic Oncology, Hunan Cancer Hospital, 283 Tongzipo Road, Yuelu District, Changsha, Hunan 410013, China

## Abstract

To evaluate the efficacy and safety of apatinib, an oral antiangiogenic drug, in patients with recurrent or refractory cervical cancer as salvage treatment, we retrospectively analyzed the medical records of recurrent or refractory cervical cancer patients admitted to the National Cancer Center/Cancer Hospital, Chinese Academy of Medical Sciences and Hunan Cancer Hospital, from October 1, 2016, to December 31, 2017. Patients who progressed within 6 months after the last treatment were given apatinib orally at a dose of 250 mg daily until disease progression or unacceptable toxicity. Twenty-nine patients were enrolled in our retrospective study. Up to February 1, 2019, the median follow-up time was 18 months. The median progression-free survival was 128 days (95% confidence interval (CI): 20–540 days), and the median overall survival was 9 months (95% CI: 4–23 months). The longest period of apatinib administration was 540 days. No complete response was observed, 5 (17.2%) patients achieved partial response, and 11 (37.9%) achieved stable disease. The objective response rate and disease control rate were 17.2% and 55.1%, respectively. The most common adverse events were hypertension (G1, 65.5%, 19/29), mucositis (G1, 55.2%, 16/29), hand-foot syndrome (G1-2, 44.8%, 13/29), and proteinuria (G1-2, 20.7%, 6/29). Grade 3 proteinuria occurred in only one patient (3.4%, 1/29). Apatinib single-agent use might be an effective and tolerable choice as salvage therapy for patients with recurrent or refractory cervical cancer.

## 1. Introduction

Cervical cancer is the most common gynecological cancer in developing countries. The incidence of cervical cancer in China is 13.3 cases per 100,000 [1]. Early stage cervical cancer can be cured by radical surgery or concurrent chemoradiation. However, recurrent and persistent cervical cancer remains incurable with limited treatment choices. Palliative chemotherapy is the current standard of care for most recurrent cervical cancer with relatively low response rate (17–30%) and short duration, especially for the patients who have received previous chemotherapy [2–5]. Most of all, a majority of patients with recurrent disease cannot tolerate the chemotherapy, and their quality of life is poor. Therefore, there is an increasing need for targeted, novel therapies with low toxicity to serve as monotherapy or adjuvant therapy for recurrent or refractory cervical cancer.

Angiogenesis is critical for the development and progression of tumor, and antiangiogenic therapy has been demonstrated to be effective in multiple cancers, including cervical cancer [5]. In cervical cancer, human papillomavirus (HPV) mediates the activation of VEGF-VEGFR pathway by promoting the expression of VEGFA, thus promoting tumor angiogenesis [6]. Many studies showed that the upregulation of VEGFR2 and the increased intratumoral microvessel density (MVD) were associated with poor prognosis and metastasis of cervical cancer [7]. This evidence indicates that antiangiogenic therapy may be applied to cervical cancer.

In the randomized, open-label, phase III trial GOG 240, bevacizumab in combination with chemotherapy showed significant improvement of overall survival (OS) for advanced cervical cancer, demonstrating its activity in this disease [5]. But the high cost of bevacizumab limits its use in developing or low-income countries. A novel oral small molecule tyrosine kinase inhibitor targeting VEGFR2, apatinib, has been demonstrated to exert an antitumor effect in a few I/II phase clinical studies of gastric cancer, NSCLC, ovarian cancer, and breast cancer [8–12]. Additionally, in a randomized, double-blinded, placebo-controlled phase III trial of apatinib, Li et al. reported that apatinib significantly improved OS and progression-free survival (PFS) of patients with stomach or gastroesophageal junction cancers [13], so it was approved and marketed for the treatment of advanced gastric cancer in China. *In vitro* and *in vivo* studies also demonstrated that apatinib showed potential efficacy for the treatment of cervical cancer [14]. Moreover, a retrospective study of apatinib for treating gynecologic malignancies including three cervical cancer patients found that apatinib might be a potential choice for cervical cancer [15]. We conducted this retrospective study to further evaluate the efficacy and safety of apatinib in patients with heavily pretreated persistent/recurrent cervical cancer.

## 2. Materials and Methods

### 2.1. Patients

Between October 1, 2016, and December 31, 2017, patients with recurrent or refractory cervical cancer at the National Cancer Center/Cancer Hospital, Chinese Academy of Medical Sciences Institution and Hunan Cancer Hospital, were enrolled. Inclusion criteria included (1) age >18 years; (2) pathologically confirmed cervical cancer; (3) progression within 6 months after the last treatment or during the last therapy including chemotherapy, radiotherapy, or chemoradiotherapy; (4) at least 1 measurable lesion according to CT or MRI; and (5) the recurrent disease not suitable for secondary surgery or radiotherapy. Patients were excluded for any of the following reasons: (1) prior antiangiogenic treatment; (2) uncontrolled hypertension; (3) proteinuria >1 g/24 hours; (4) vaginal bleeding; and (5) arterial embolism or thrombosis. Informed consent was obtained from all the patients before the start of apatinib therapy. This study was approved by the Institutional Review Board of Cancer Hospital, Chinese Academy of Medical Sciences. Data including demographic information, clinical-pathologic information, treatment history, disease status, survival status, and adverse events were collected for analysis.

### 2.2. Treatment

Apatinib was administered at a daily dose of 250 mg p.o., for a cycle of 4 weeks. Treatment interruption up to 14 days was allowed if intolerable toxicities developed. Patients discontinued oral administration of apatinib if they experienced disease progression or unacceptable toxicity after the dose interruption of >14 days.

### 2.3. Efficacy and Safety Assessments

Disease status was assessed by CT or MRI every 8 weeks. The SCC antigen, a widely used tumor marker for squamous cell carcinoma, was tested at every cycle. If SCC doubled compared to the baseline, imaging was warranted to determine progression. The tumor response was evaluated according to Response Evaluation Criteria in Solid Tumors 1.1 criteria [16]. The objective response rate (ORR) was defined as the sum of complete response (CR) and partial response (PR). The disease control rate (DCR) was defined as the addition of CR, PR, and stable disease (SD). PFS was defined as the period from the beginning of apatinib treatment to disease progression or death. OS was defined as the duration from the beginning of apatinib treatment to the date of death for any reason or to the last date of follow-up, whichever came first. Blood pressure was monitored once every morning. Hematology, serum chemistry, routine urine examination, physical examination, and 12-lead electrocardiogram were assessed every 4 weeks. Adverse events were evaluated by the National Cancer Institute Common Terminology Criteria for Adverse Events version 5.0 [17].

### 2.4. Statistical Analysis

Statistical analyses were performed using SPSS software, version 22.0 (SPSS Inc., Chicago, IL, USA). Continuous data were expressed as the median (range) and categorical variables as percentages. PFS and OS were estimated by the Kaplan–Meier method.

## 3. Results

### 3.1. Patient Characteristics

A total of 29 patients were retrospectively enrolled in the study (Table 1). The median age was 48 years (range 38–61 years). Twenty-five (86.2%) patients were diagnosed with cervical squamous cell carcinoma, three (10.3%) patients with cervical small cell carcinoma, and one (3.4%) patient with cervical adenocarcinoma. The primary treatment was radical surgery ± adjuvant therapy for 9 (31.0%) patients with stage IB2-IIA2 disease, while 20 (69.0%) patients with stage IIB-IVB disease underwent concurrent radiochemotherapy. No patient had received prior antiangiogenic therapy. Thirteen (44.8%) patients exhibited disease progression during previous treatment, and the other 16 (55.2%) recurred within 6 months after the previous treatment. The median number of prior chemotherapy regimens was 3 (range 1 to 5). The main regimens of prior chemotherapy included cisplatin, 5-FU + cisplatin, paclitaxel + cisplatin, and topotecan. The median treatment-free interval before the start of apatinib was 2 months. Seven (24.1%) patients exhibited recurrent disease within the previously irradiated field, 17 (58.6%) patients exhibited outside of the previously irradiated field or without previous radiation history, and 5 (17.2%) patients exhibited multiple recurrences both within and outside of the irradiated field.

### 3.2. Efficacy

**Figure 1:**
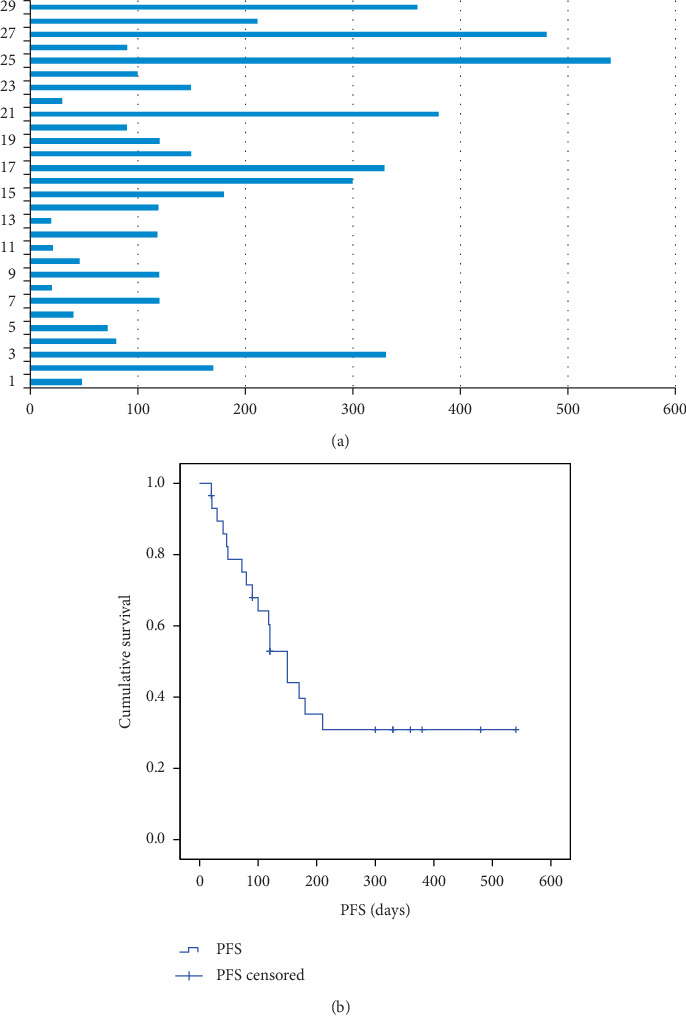
(a) Duration of apatinib treatment in patients (days). (b) Kaplan–Meier plot for PFS.

**Figure 2:**
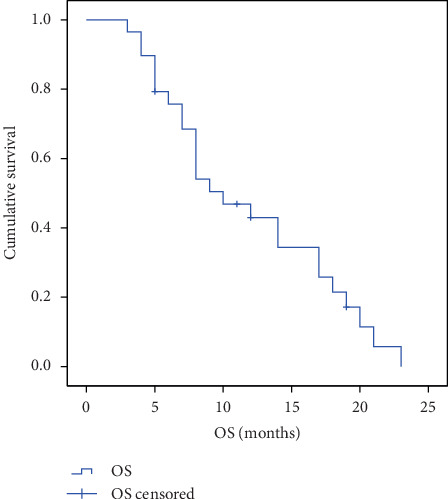
Kaplan–Meier plot for OS.

At the time of the last follow-up on February 1, 2019, with a median follow-up time of 18.0 months, all patients had discontinued administration, and the longest time of apatinib administration was 540 days (Figure 1(a)). Twenty-six (89.7%) patients discontinued treatment due to disease progression, and 3 patients with stable disease discontinued treatment due to either appendectomy, pneumonia fever, or grade 3 proteinuria, respectively. At the end of the follow-up, 25 patients had died, 2 patients were still alive, and the 2 remaining patients have lost to follow-up at 3 and 7 months after discontinuation of apatinib, respectively. The median PFS was 128 days (95% CI: 20–540 days) (Figure 1(b)), and the median OS was 9 months (95% CI: 4–23 months) (Figure 2). None of the patients achieved CR. A total of 5 patients (17.2%, 5/29) achieved PR, and 11 patients (37.9%, 11/29) achieved SD. ORR and DCR were 17.2% and 55.2% (16/29), respectively.

### 3.3. Safety

Treatment-related adverse events (AEs) are summarized in Table 2. The most common AEs were hypertension (G1, 65.5%, 19/29), mucositis (G1, 55.2%, 16/29), hand-foot syndrome (G1, 41.4%, 12/29; G2, 3.4%, 1/29), neutropenia (G1-2, 27.6%, 8/29), and proteinuria (G1-2, 20.7%, 6/29). Grade 3 proteinuria occurred in only one patient (3.4%, 1/29) after administration of apatinib for 118 days with full recovery after supportive treatment within 2 weeks. No grade 4 AE was observed in the study.

## 4. Discussion

Patients with recurrent/refractory cervical cancer after failing multiple-line treatment show very poor response to therapy and are intolerant to chemotherapy. Treatment of such patients remains a crucial challenge in cervical cancer management. Various single-agent or combination therapies have been evaluated in this particular patient population, yet disease outcome is disappointing, often accompanied by compromised quality of life. Therefore, the identification of a more effective low-toxic therapy is paramount.

In the past two decades, antiangiogenic agents have exhibited promising antitumor efficacy in multiple solid tumors such as colorectal cancer and ovarian cancer. In 2014, the GOG 240 trial, for the first time, established the critical role of an antiangiogenic agent in cervical cancer treatment combining bevacizumab with chemotherapy [5]. Meanwhile, various antiangiogenetic agents such as apatinib are under investigation in multiple solid tumors including cervical cancer.

Apatinib, as a novel, oral, highly selective tyrosine kinase inhibitor, blocks downstream signaling of VEGFR2 by targeting its ATP-binding site. Pharmacodynamics studies showed that apatinib could suppress the tyrosine kinase activity of VEGFR, block the VEGF-induced signal transduction, and result in the inhibition of tumor angiogenesis [18]. Preclinical data also showed that apatinib effectively inhibited proliferation, migration, and tube formation of human umbilical vein endothelial cells. Subsequently, the use of animal models confirmed that apatinib blocked the budding of rat aortic ring and inhibited the growth of xenograft tumors, either alone or in combination with chemotherapeutic drugs [19, 20].

Apatinib has been rarely reported for the treatment of cervical cancer. In two retrospective studies with a limited number of patients with metastatic or recurrent cervical cancers, apatinib showed potential efficacy [21, 22] with a median PFS of 3.0 to 7.0 months, a median OS of 7.0 to 16.0 months, an ORR of 15.4 to 16.7%, and a DCR of 57.7 to 67.7%. In the present study, twenty-nine recurrent or refractory cervical cancer patients received apatinib. We reported that the PFS was 128 days, OS was 9 months, ORR was 17.2%, and DCR was 55.2%, which was comparable with the results of the above two studies. Currently, cisplatin is one of the most effective cytotoxic agents for the treatment of recurrent or metastatic cervical cancer, with an ORR ranging from 20–30% and a median OS of 6–9 months [2, 3, 23]. Therefore, apatinib might be a valuable choice for recurrent cervical cancer especially considering the toxicity associated with chemotherapy.

The common adverse events of apatinib, including hypertension, mucositis, hand-foot syndrome, and proteinuria, seemed to be similar to those observed with other antiangiogenic agents [24]. Our study further investigated apatinib safety profile at a lower dose (250 mg) than that previously reported (500 mg) in consideration of multiple lines of pretreatment, poor bone marrow reserve, and weak physical conditions of this patient population. Notably, in the studies reported by Yu et al. and Chen et al., dose reduction was as high as 53.8% and 66.7% when administrating apatinib at a daily dose of 500 mg [21, 22]. Our results successfully demonstrated an outstanding advantage of a low-dose (250 mg) regimen with only one grade 3 AE with proteinuria who fully recovered within 2 weeks and no occurrence of grade 4 AE. Most of AEs were relatively mild (grade 1-2) and manageable, thus not requiring treatment discontinuation. Moreover, our current study with low-dose apatinib reached comparable PFS and OS to those of Yu's and Chen's studies. In addition, low-dose apatinib allowed increased treatment duration as described in the present study, i.e., a maximum of 540 days. The sustainability of apatinib might bring further benefits to declining patients with poor outcome. Therefore, low-dose apatinib might be a promising alternative treatment for recurrent or refractory cervical cancer patients. The limitations of the current study rely on the nature of the retrospective study and the limited sample size.

## 5. Conclusions

Apatinib had potential efficacy in heavily pretreated recurrent and refractory cervical cancer with manageable toxicities. A well-designed, prospective, and controlled study is warranted to validate the efficacy and safety of low-dose apatinib in this patient population.

## Figures and Tables

**Table 1:** Patient's clinical-pathologic characteristics.

Characteristics	
Age (years)	48 (30–63)
FIGO stage	
IB2-IIA2	9 (31.0%)
IIB-IVB	20 (69.0%)
Histology	
Cervical squamous cell carcinoma	25 (86.2%)
Cervical adenocarcinoma	1 (3.4%)
Cervical small cell cancer	3 (10.3%)
Sites of recurrence	
Within the irradiated field	7 (24.1%)
Outside the irradiated field	17 (58.6%)
Within and outside the irradiated field	5 (17.2%)
Median number of previous chemotherapy regimens	3 (1–5)
Median interval between last treatment and disease progression (months)	2 (0–6)

FIGO: the International Federation of Gynecology and Obstetrics.

**Table 2:** Analysis of patient safety.

Adverse event	*N* (%)
G1 hypertension	19 (65.5)
G1 mucositis	16 (55.2)
G1-2 hand-foot syndrome	13 (44.8)
G1-2 proteinuria	6 (20.7)
G3 proteinuria	1 (3.4)
G4	0 (0)

G: grade.

## Data Availability

The data used to support the findings of this study are available from the first author and the corresponding author upon request.
